# P-46. Characterization of Antibiotic Resistance Mechanisms in Bacteremia and Potential Novel Therapeutic Approaches at Rajshahi Medical College

**DOI:** 10.1093/ofid/ofaf695.275

**Published:** 2026-01-11

**Authors:** Abu Hena Mostafa Kamal, Wahida Khatun

**Affiliations:** Rajshahi Medical College & Hospital, Rajshahi-6000, Rajshahi, Bangladesh; RMCH,Rajshahi, Rajshahi, Rajshahi, Bangladesh

## Abstract

**Background:**

Bacteremia caused by multidrug-resistant (MDR) bacteria presents a significant challenge to healthcare systems worldwide, particularly in intensive care units (ICU). At Rajshahi Medical College, the burden of antibiotic-resistant infections is increasing.

**Methods:**

A prospective observational study was conducted between June 2023 and June 2024, including 264 ICU patients with blood cultures positive for bacterial pathogens. Identification and antibiotic susceptibility testing were performed using disk diffusion and VITEK-2 systems. Resistance mechanisms, including β-lactamase production, efflux pump activity, and target-site mutations, were evaluated through PCR and sequencing. A total of 12 different antibiotics were tested, and resistance rates were analyzed. Statistical analyses were performed using SPSS 23, with p-values < 0.05 indicating significance. Standard deviations (SD) for resistance rates were calculated to assess variability.

**Results:**

Among the 264 isolates, 99 (37.5%) demonstrated resistance to at least three antibiotic classes, classified as multidrug-resistant (MDR). *Staphylococcus aureus* accounted for 23.5%, and *Escherichia coli* contributed to 18.7% of the resistant strains. The overall resistance rate to methicillin for *S. aureus* was 82%, with a standard deviation of ±5%. Extended-spectrum β-lactamase (ESBL) production was present in 18.7% of *E. coli* isolates, with a resistance rate of 72% to cephalosporins and a standard deviation of ±3%. A significant correlation was observed between prolonged antibiotic exposure ( >7 days) and increased resistance (p< 0.01), with resistance rates escalating by 15%. Efflux pump activity was detected in 32% of isolates, correlating with higher resistance to fluoroquinolones (p< 0.05). In vitro testing with antimicrobial peptides demonstrated a 65% reduction in bacterial load (SD ±4%), and bacteriophage therapy trials reduced bacterial growth by 58% (SD ±6%).

**Conclusion:**

Antibiotic resistance in bacteremia is prevalent in ICU patients at Rajshahi Medical College. The study highlights the need for novel therapeutic interventions, such as antimicrobial peptides and bacteriophage therapy, showing promise in reducing bacterial loads.

**Disclosures:**

All Authors: No reported disclosures

